# Editorial for the Special Edition of Advanced Prostate Cancer: From Bench to Bedside

**DOI:** 10.3390/cancers15041247

**Published:** 2023-02-16

**Authors:** Fred Saad

**Affiliations:** Division of Urology, University of Montreal Hospital Center (CHUM), Montreal, QC H2X 3E4, Canada; fred.saad@umontreal.ca

Prostate cancer is generally viewed as a slow-growing unaggressive cancer, yet it is one of the most commonly diagnosed cancers and a leading cause of morbidity and mortality in men around the world. Over the last few years, the management of advanced prostate cancer has evolved tremendously, leading to new therapeutic options that have helped to improve survival and quality of life. All of this success is due to the intensive research that has focused on preclinical models and transitioned through the phases of clinical research to eventually lead to what we now have to offer. This Special Edition had the objective of reviewing some of the therapeutic options presently available and how best to use these options in advanced/metastatic castration-sensitive, as well as castration-resistant, prostate cancer. This Special Issue also addressed some of the ongoing basic and clinical research that may lead to new therapies and also how to better use the options we have at our disposal, whether it be in terms of timing, combination, or sequencing. Also of interest is the exponential growth of molecular imaging and radioligand therapy over the past few years. Highlighting work that integrates predictive, prognostic, and genomic biomarkers is becoming critically important if we are to optimally use the available therapeutics and to help develop new effective options for the future.

I am very proud that the Special Edition entitled Advanced Prostate Cancer: From Bench to Bedside was successful in regrouping several extremely interesting and informative articles. I thank the outstanding group of authors for their contributions to this Special Edition. I hope you will find these articles both useful and inspiring for future studies in prostate cancer. Finally, it is with much pride that I realize that at the time I write this editorial, these articles have had a total of almost 25,000 views.

It is with pleasure that I provide a very brief summary of the 12 articles in this Special Issue and encourage you to view these excellent papers in detail.

Adediran et al. published the paper entitled **Co-Targeting of ErbB Receptors and the PI3K/AKT Axis in Androgen-Independent Taxane-Sensitive and Taxane-Resistant Human Prostate Cancer Cells** [1] and addressed the important issue of creating novel therapeutic targets in patients with advanced chemo-sensitive and chemo-resistant tumors. They convincingly conclude that co-targeting AKT with ErbB, and possibly other partners, may be a useful strategy to further explore the potential therapeutic effect in advanced PCa.

The article by Dariane et al. entitled **High Keratin-7 Expression in Benign Peri-Tumoral Prostatic Glands Is Predictive of Bone Metastasis Onset and Prostate Cancer-Specific Mortality** [2] provides details on a potential biomarker that could be useful in predicting the development of bone metastases and early death in patients with prostate cancer. They found that high KRT7 expression in benign glands is an independent biomarker of MFS and CSS, and its expression is lost in tumoral cells. They conclude that more work is warranted, and this may eventually lead to a novel therapeutic approach to improve the outcome of these patients.

The article by Carr et al. entitled **Homologous Recombination Repair Gene Mutation Characterization by Liquid Biopsy: A Phase II Trial of Olaparib and Abiraterone in Metastatic Castrate-Resistant Prostate Cancer** [3] addresses the important issue of identifying homologous recombination repair gene mutation (HRRm) beyond tissue testing, which is challenging and often impossible. The authors report their findings of a phase 2 randomized trial and showed that germline and plasma testing increased the HRRm characterized population from 27% to 68% of 142 randomized patients. The authors conclude that their results confirm the value of plasma testing for HRRm status when there is insufficient high-quality tissue for multi-gene molecular testing. Importantly, this strategy is being tested in several phase 3 studies regarding the role of PARP inhibitors in prostate cancer.

The article by Rounds et al. entitled **Glyoxalase 1 Expression as a Novel Diagnostic Marker of High-Grade Prostatic Intraepithelial Neoplasia in Prostate Cancer** [4] proposes a biomarker that appears to be capable of identifying prostatic intraepithelial neoplasia (HGPIN) and the ability to change the paradigm by which the pathological assessment of HGPIN status solely depends on morphological features. The results of their study identified upregulated GLO1 expression as a molecular hallmark of HGPIN lesions that were detectable by immunohistochemical analysis and may serve as a novel diagnostic marker that can identify this precancerous lesion. 

Mosillo et al. published a paper entitled **Targeted Approaches in Metastatic Castration-Resistant Prostate Cancer: Which Data?** [5], which reviews the important topic of the complex pathways underlying the inherited and acquired mechanisms of resistance to the available treatments. They rightly propose that a better understanding of these pathways may lead to significant improvements in survival by providing innovative therapeutic targets. They also provide an excellent overview of the recent progress in novel targeted therapies and near-future directions. After 60 years in which castration was the only treatment offered for advanced prostate cancer, the field has drastically expanded with new therapeutic options over the last decade and continues to move ahead, which is excellent news for physicians and patients.

Beyond the central role of effective systemic therapy for metastatic prostate cancer, the question of metastasis-directed therapy continues to be both attractive and controversial. Juan et al. provide an excellent summary of the available data in their article **Where Do We Stand in the Management of Oligometastatic Prostate Cancer? A Comprehensive Review** [6]. They propose that OMPC is a unique clinical state with inherently indolent tumor biology that is susceptible to multi-disciplinary treatment (MDT). They conclude, although this has still not been fully established, that multi-modal management is necessary to improve patient outcomes, due to the combination of the available therapies, such as local therapy of primary tumors, metastasis-directed therapy or systemic therapy, to reduce tumor load and prevent further disease progression. They do, however, conclude that additional prospective data are needed to select the patients that are most likely to benefit from a given therapeutic approach. If this can be proven, even in a subset of patients, it would be extremely important, since it would have the potential to cure patients that would be considered as incurable.

Clark et al. provide an insightful review in their paper entitled **Clinical Management of Prostate Cancer in High-Risk Genetic Mutation Carriers** [7]. They rightly state that it is critical to identify individuals who are at high risk for disease progression and death and that germline genetic differences are increasingly recognized as contributing factors to the risk of lethal prostate cancer. They also review the management options for men with high-risk germline mutations, but acknowledge that the overall quality of the evidence is low. Since this remains a challenge for one of the most common and lethal cancers worldwide, they conclude that there is an immediate need for further research and the development of consensus guidelines to guide clinical practice for these individuals. 

Rao et al. also provide an excellent review that addresses the rationale of combining the inhibition of the androgen receptor and PARP in their review paper **Co-Inhibition of Androgen Receptor and PARP as a Novel Treatment Paradigm in Prostate Cancer—Where Are We Now?** [8]. They summarize the growing body of preclinical and clinical data, which show that the co-inhibition of AR and PARP induces synthetic lethality and could be a promising therapy for patients without any DDR alterations. They also review the limitations of novel anti-androgen monotherapy, the most-commonly used systemic therapeutic treatment in patients with advanced prostate cancer. They effectively review the mechanistic rationale for synthetic lethality induced by the co-inhibition of AR and PARP and the available clinical data that have led to the global development of a number of these AR and PARP combination therapies, and how this may impact patient care in the near future.

Poon et al. tackle the very important issue of ethnic variability related to outcome form treatment in prostate cancer in their review entitled **Ethnic Pharmacogenomic Differences in the Management of Asian Patients with Metastatic Prostate Cancer** [9]. The authors acknowledge the unprecedented advancement in systemic therapies that has resulted in the overall improvement of survival rates in men with advanced prostate cancer. They address the ongoing controversies over how best to treat these patients, but also indicate the importance of the recognition that ethnicity may also influence prognosis and outcomes. Their review discusses recent evidence for the impacts of Asian ethnicity specifically, which includes environmental, sociocultural, and genetic factors, on the approach to the pharmacological management of metastatic prostate cancer. They conclude that there are clear inter-ethnic differences in drug tolerability, serious adverse events (AEs), and genetic heterogeneity. They conclude that these findings must be considered when administering and scheduling treatment, as well as designing future precision studies on prostate cancer.

Westaby et al. review a novel approach to eventually improving or adding to the therapeutic treatment options for prostate cancer in their article **Targeting the Intrinsic Apoptosis Pathway: A Window of Opportunity for Prostate Cancer** [10]. While they acknowledge the advancements in therapeutic options over the last few years, they propose that the idea of inducing cancer cell death via apoptosis has long been an attractive goal in the treatment of cancer. The review provides an up-to-date overview of cell death and apoptosis, specifically focusing on the associated intrinsic pathway. It summarizes the latest approaches for targeting the intrinsic apoptosis pathway with BH3 mimetics, which have shown promising efficacy for hematological malignancies. They provide insight into how these strategies may be leveraged to treat prostate cancer.

Burgess et al. provide a comprehensive and updated review of the management of high-risk prostate cancer in their paper **A Review on the Current Treatment Paradigm in High-Risk Prostate Cancer** [11]. While high-risk prostate cancer is traditionally treated with a combination of radiotherapy (RT) and androgen deprivation therapy (ADT), they provide insight into the recent advancements in systemic treatment and radiotherapy that have widened the spectrum of treatment for this patient population. The use of image guidance and intensity modulation, as well as the incorporation of brachytherapy, has led to safe radiotherapy dose escalation with reduced risk of recurrence. They also provide some data on the emerging evidence that has redefined the role of surgery in this cohort. They review some contemporary clinical trials that have identified new systemic therapy options in high-risk prostate cancer patients. Finally, the paper provides insight into the introduction of new imaging modalities, including multi-parametric MRI and molecular imaging and genomic classifiers that are slowly being integrated in patient selection, risk stratification, and treatment tailoring.

Zhang et al. review the growing enthusiasm and use of radioligand therapy in prostate cancer in their review **PSMA Theranostics: Current Landscape and Future Outlook** [12]. They provide a comprehensive review of the prostate-specific membrane antigen (PSMA) as a promising novel molecular target for imaging diagnostics and therapeutics (theranostics). They provide a summary of the evolving evidence for various PSMA-targeted radiopharmaceutical agents in the treatment paradigm for prostate cancer, as well as combination treatment strategies with other targeted therapies and immunotherapies. They also discuss the evidence of PSMA and fluorodeoxyglucose (FDG) PET/CT as a predictive biomarker for PSMA radioligand therapy. Overall, the review provides a contemporary and future landscape of theranostic applications in prostate cancer, with a focus on PSMA ligands. Given the emerging use of this therapeutic approach in advanced prostate cancer, they emphasize the importance of integrating nuclear medicine physicians into the multi-disciplinary teams in clinics.

In conclusion, these papers provide readers with a glimpse into the challenges faced when dealing with prostate cancer, while providing optimism that through research and multi-disciplinary collaboration, great progress has already been made in both our understanding and the treatment of advanced prostate cancer.

## References

[1] Adediran S., Wang L., Khan M.A., Guang W., Fan X., Dan H., Qi J., Jay S.M., Carrier F., Hussain A. (2022). Co-Targeting ErbB Receptors and the PI3K/AKT Axis in Androgen-Independent Taxane-Sensitive and Taxane-Resistant Human Prostate Cancer Cells. Cancers.

[2] Dariane C., Clairefond S., Péant B., Communal L., Thian Z., Ouellet V., Trudel D., Benzerdjeb N., Azzi F., Méjean A. (2022). High Keratin-7 Expression in Benign Peri-Tumoral Prostatic Glands Is Predictive of Bone Metastasis Onset and Prostate Cancer-Specific Mortality. Cancers.

[3] Carr T.H., Adelman C., Barnicle A., Kozarewa I., Luke S., Lai Z., Hollis S., Dougherty B., Harrington E.A., Kang J. (2021). Homologous Recombination Repair Gene Mutation Characterization by Liquid Biopsy: A Phase II Trial of Olaparib and Abiraterone in Metastatic Castrate-Resistant Prostate Cancer. Cancers.

[4] Rounds L., Nagle R.B., Muranyi A., Jandova J., Gill S., Vela E., Wondrak G.T. (2021). Glyoxalase 1 Expression as a Novel Diagnostic Marker of High-Grade Prostatic Intraepithelial Neoplasia in Prostate Cancer. Cancers.

[5] Mosillo C., Calandrella M.L., Caserta C., Macrini S., Guida A., Sirgiovanni G., Bracarda S. (2022). Targeted Approaches in Metastatic Castration-Resistant Prostate Cancer: Which Data?. Cancers.

[6] Juan G.R., Laura F.H., Javier P.V., Natalia V.C., Mᵃ Isabel G.R., Enrique R.G., José Luis S.P., Pablo A.L., Noelia S.S., Roser V.D. (2022). Where Do We Stand in the Management of Oligometastatic Prostate Cancer? A Comprehensive Review. Cancers.

[7] Clark R., Herrera-Caceres J., Kenk M., Fleshner N. (2022). Clinical Management of Prostate Cancer in High-Risk Genetic Mutation Carriers. Cancers.

[8] Rao A., Moka N., Hamstra D.A., Ryan C.J. (2022). Co-Inhibition of Androgen Receptor and PARP as a Novel Treatment Paradigm in Prostate Cancer—Where Are We Now?. Cancers.

[9] Poon D.M.C., Chan K., Chan T., Cheung F.-Y., Lam D., Lam M., Law K.-S., Lee C., Lee E.K.C., Leung A. (2022). Ethnic Pharmacogenomic Differences in the Management of Asian Patients with Metastatic Prostate Cancer. Cancers.

[10] Westaby D., Jimenez-Vacas J.M., Padilha A., Varkaris A., Balk S.P., de Bono J.S., Sharp A. (2022). Targeting the Intrinsic Apoptosis Pathway: A Window of Opportunity for Prostate Cancer. Cancers.

[11] Burgess L., Roy S., Morgan S., Malone S. (2021). A Review on the Current Treatment Paradigm in High-Risk Prostate Cancer. Cancers.

[12] Zhang H., Koumna S., Pouliot F., Beauregard J.-M., Kolinsky M. (2021). PSMA Theranostics: Current Landscape and Future Outlook. Cancers.

